# Mechanical regulation of calcium signaling of HL-60 on P-selectin under flow

**DOI:** 10.1186/s12938-016-0271-1

**Published:** 2016-12-28

**Authors:** Bing Huang, Yingchen Ling, Jiangguo Lin, Ying Fang, Jianhua Wu

**Affiliations:** School of Bioscience & Bioengineering, South China University of Technology, Guangzhou, 510006 China

**Keywords:** HL-60 cell line, P-selectin, Calcium signaling, Shear stress, Flow chamber

## Abstract

**Background:**

Binding of P-selectin to P-selectin glycoprotein ligand-1 (PSGL-1) makes neutrophils roll on and adhere to inflammatory site. Intracellular calcium bursting of adhered neutrophils is a key event for subsequent arresting firmly at and migrating into the injured tissue. But, it remains unclear how the cytoplasmic calcium signaling of the cells were modulated by the fluid shear stress. Here, we focus on mechanical regulation of P-selectin-induced calcium signaling of neutrophil-like HL-60 cells under flow.

**Methods:**

HL-60 cells were loaded with Fluo-4 AM for fluorescent detection of intracellular calcium ion, and then perfused over P-selectin-coated bottom of parallel-plate flow chamber. The intracellular calcium concentration of firmly adhered cell under flow was observed in real time by fluorescence microscopy.

**Results:**

Force triggered, enhanced and quickened cytoplasmic calcium bursting of HL-60 on P-selectin. This force-dependent calcium signaling was induced by the immobilized P-selectin coated on substrates in absence of chemokine. Increasing of both shear stress and P-selectin concentration made the calcium signaling intensive, through quickening the cytosolic calcium release and upregulating both probability and peak level of calcium signaling.

**Conclusions:**

Immobilized P-selectin-induced calcium signaling of HL-60 cells is P-selectin concentration- and mechanical force-dependent. The higher both the P-selectin concentration and the external force on cell, the more intensive the calcium signaling. It might provide a novel insight into the mechano-chemical regulation mechanism for intracellular signaling pathways induced by adhesion molecules.

## Background

Recruitment of polymorphonuclear leukocytes (PMNs) to vessel endothelium of injury or infection is essential for the inflammatory response process [1–4]. PMN recruitment is a multistep process, including events such as rolling, activation, adhesion and transmigration, and supported by specific adhesion molecules, such as selectins and integrins, which coordinate the interactions between leukocytes and endothelial cells [1–4]. In this process, neutrophils activation is essential for innate immune defense against infection and injury [2].

P-selectin, as one of the main adhesion molecules expressed constitutively at the surface of activated vascular endothelial cells [5], binds primarily to P-selectin glycoprotein ligand-1 (PSGL-1) on neutrophils to support cells rolling and adhesion [6, 7]. Selectins binding with PSGL-1 also contributes to β2 integrin activation, which requires sufficient cytosolic calcium [8]. Cytosolic calcium is the most ubiquitous second messenger involved in a variety of intracellular signaling pathways for most cellular functions [9]. Increasing the concentration of cytoplasmic free calcium induces cell activation, triggers superoxide generation [10], enzyme secretion [11], actin gel-sol transition [12] and locomotion [13, 14], and is a sensitive indicator of the level of neutrophil activation during recruitment to inflammatory sites under shear stress [8, 15]. The oxidative stress-induced calcium release from internal stores could lead to mitochondrial membrane depolarization, sudden caspase-9 and -3 activation of human promyelocytic leukemia cell HL-60 [16]. Chemokine combining with GPCR can induce intracellular calcium upregulation in neutrophils [17–19], and selectins engagement can induce calcium flux in neutrophils under flow condition in absence of chemokine [8, 20].

Besides, it was demonstrated that immune functions of leukocytes are influenced by not only chemical but also mechanical factors [21]. External force was an important regulator of interactions between PSGL-1 and (P-, E-, and L-) selectins to mediate HL-60 rolling and adhesion [7, 22], and influence calcium signaling in T cell mediated by TCR/MHC-peptide [23]. Calcium concentration of neutrophils rolling on E-selectin in presence of chemokine increased much in higher shear flow (2 dyn/cm^2^) but did not in lower shear flow (0.2 dyn/cm^2^) [8], and T-lymphocyte calcium signaling is regulated by mechanical force too [23]. However, it remains unclear whether P-selectin induces calcium signaling in neutrophils under flows or not.

Human leukaemic HL-60 cells, the neutrophilic promyelocytes, are widely used for studying their rolling behaviour on selectins [22, 24], signal transduction mechanisms [25, 26] and acute promyelocytic leukemia differentiation therapy [27, 28], because they can be easily got and modified by gene engineering [29]. In the present work, we examined intercellular calcium signaling of HL-60 on P-selectin with the parallel-plate flow chamber and fluorescence microscope techniques. The events of P-selectin-induced calcium signaling under various wall shear stress were characterized by activation ratio, peak intensity and delay time or latency of calcium bursting. The results indicated that force triggered calcium signaling of firmly adhered HL-60 cells on immobilized P-selectin in absence of chemokine under flow, and the P-selectin-induced calcium signaling of HL-60 is P-selectin concentration- and force-dependent, showing a novel sight for the cellular physiologic process at molecular level.

## Methods

### Proteins and cells

Human promyelocytic leukemia HL-60 cells (Cell Bank of Chinese Academy of Sciences, Shanghai, China), constitutively expressed PSGL-1 as a ligand for P-selectin, were maintained in RPMI-1640 medium supplemented with 10% fetal bovine serum (FBS), 10 mg/mL streptomycin and 100 units/mL penicillin at 37 °C in a humidified atmosphere of 5% CO_2_ in air. RPMI-1640 medium, FBS and BSA were purchased from Sigma Chemical Co. (St Louis, MO, USA). Streptomycin, penicillin and phosphate buffer saline (PBS) were obtained from Gibco BRL (Grand Island, NY). Recombinant Human P-Selectin/CD62P Fc Chimera Protein (R&D Systems, Minneapolis, MN) is a disulfide-linked homodimer, containing the Fc moiety of human IgG and the extracellular domain of human P-selectin.

### Functionalization of flow chamber

Flow chamber (length × width × height = 2 × 0.25 × 0.0127 cm^3^) was functionalized through the procedure described in our previous work [7, 22]. Dry powder of P-selectin was dissolved by PBS. P-selectin was absorbed directly onto a cover slide (Fisher Scientific, Pittsburgh, PA) by adding 20 µL P-selectin solution into a coating region (2.5 × 5 mm^2^), which was held by a hollowed silicon gasket and marked in the cover slide center, and incubated overnight at 4 °C. After removal of excessive unabsorbed P-selectin, polystyrene petri dishes were washed with PBS containing 1% BSA 3 times, and incubated in the same solution for 1 h at room temperature to block nonspecific cell adhesion. The site density of P-selectin on substrate was determined by ^125^I radioiodination method [30, 31]. Mouse anti-human P-selectin mAB 9E1 (R&D system, MN) were labeled by Pierce Iodination Kit (Thermo Fisher, IL) and purified by Sephadex G-25 column. The ^125^I labeled antibody was then added onto the P-selectin coated on substrate. The radiation intensity of ^125^I was detected by GE infinia Hawkeye 4 SPECT (GE Healthcare) after removing excessive antibody. The site densities of 0.1, 1.0 and 10 µg/mL P-selectin absorbed on polystyrene petri dish were determined to be 21, 208 and 1359 ^#^/μm^2^. These P-selectin densities were selected to support the firm adhesion of HL-60 on the substrates.

### Loading with calcium sensitive dye

Relative intracellular calcium levels were assessed by using the calcium indicator Fluo-4 acetoxymethyl (AM) ester, which was obtained from Invitrogen Life Technologies (Grand Island, NY, USA). 1 μM Fluo-4 AM was loaded into cells by incubating cells at a concentration of 1 × 10^6^/mL for 30 min at 37 °C in loading buffer (20 mM HEPES, 20 mM Glucose and 1% BSA in PBS). After 10 min centrifugation at 400×*g*, cells were re-suspended in loading buffer and incubated further for 30 min at 37 °C until use, which allowed complete de-esterification of intracellular AM ester.

### Cell adhesion and calcium signaling assays

The 10^6^/mL HL-60 cell suspensions were perfused into flow chamber with substrates of blank (without BSA and P-selectin) or P-selectin (0, 0.1, 1, 10 μg/mL with 1% BSA) at 2 dyn/cm^2^ for 7 min. We tracked about 50 cells rolling on the bottom in the direction of flow at each experiment, and defined a firm adhesion event as the cell displacement is smaller than 10 μm (approximately a cell diameter) in observation time of 1 min.

The labeled cells were re-suspended at a concentration of 1 × 10^6^/mL in buffer (110 mM NaCl, 10 mM KCl, 10 mM Glucose, 30 mM HEPES, 1.5 mM CaCl_2_, 1% BSA (w/v), 12% Ficoll (w/v) and pH 7.35). With a syringe pump (Harvard PHD22/2000, Harvard Apparatus, Holliston, MA), HL-60 suspension was perfused over P-selectin in a parallel-plate flow chamber at different wall shear stresses (0.2, 0.6 and 2 dyn/cm^2^) [7, 22]. Images containing fluorescence of firmly adhered cells were acquired by the QImaging 2000R camera coupled to the Zeiss Axio microscope. Images were analyzed by using Image Pro Plus v6.0 and Microsoft Excel 2013. The fluorescence intensity of a cell was normalized by FI_N_ = (FI_C_−FI_B_)/FI_B_, where FI_N_ is the normalized cell fluorescence intensity, FI_C_ is the mean fluorescence intensity over the cell, and FI_B_, the fluorescence intensity of background, is the mean of four fluorescence intensities sampled from four equidistant round domains (of 36π µm^2^) rung the cell with a distance of 24 µm.

### Statistics methods

The data were compared by one-way analysis of variance (ANOVA) and Student’s t test, and considered statistically significant if *p* < 0.05.

## Result

### Calcium bursting of HL-60 on P-selectin under flow

**Fig. 1:**
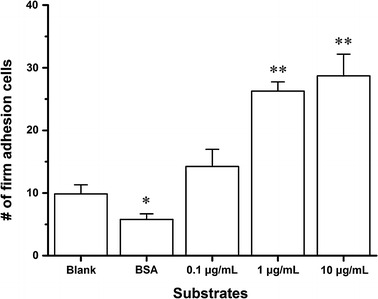
Number of firmly adhered HL-60 on substrates with five different treatments under shear stress of 2 dyn/cm^2^. The substrate were treated by coating with blank, BSA only or plus P-selectin (0.1, 1 and 10 μg/mL). The data represent the mean plus SEM from three independent experiments. The significant level of difference from blank substrate group was shown by *p* value, * for *p* < 0.05 and ** for *p* < 0.01

**Fig. 2:**
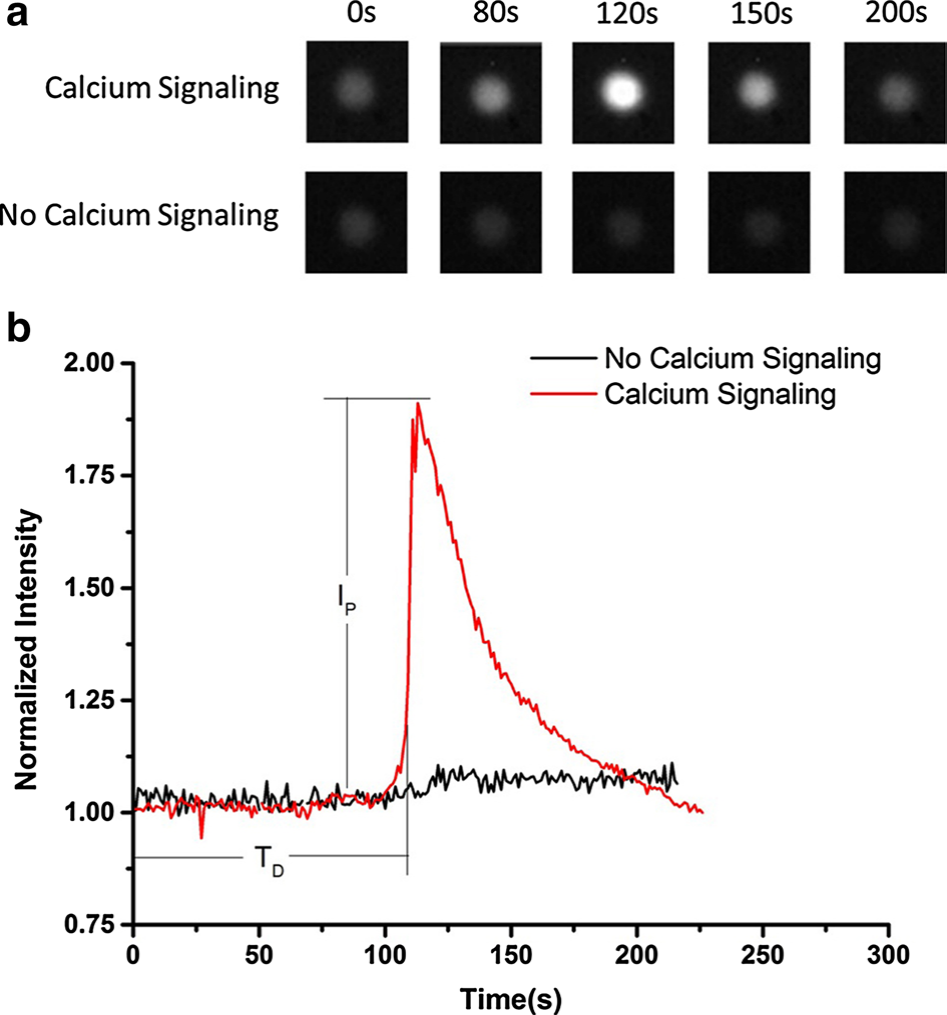
Calcium bursting of firmly adhered HL-60 on P-selectin under wall shear stress. **a** Two series of typical fluorescence images of firmly adhered HL-60 cells on P-selectin at different times, and **b** the time-course of the normalized fluorescence intensity of the cells over the observation time. Here *red* and *black* expressed the cases with or without external force (wall shear stress) of 2 dyn/cm^2^ on cells

To investigate P-selectin-mediated calcium signaling of HL-60 under flow, HL-60 cells were perfused over substrates coated with or without P-selectin under wall shear stresses of 0.2, 0.6 and 2 dyn/cm^2^, the events of firm adhesion were counted one by one, and the calcium bursting of the firmly adhered HL-60 cells were examined and recorded by the fluorescence camera (“Methods” section). The adhesion events were specific for P-selectins because these events occurred rarely for substrates treated with nothing or 1% BSA only in comparison with those for substrates coated with 1% BSA plus 0.1, 1 or 10 μg/mL P-selectin (Fig. 1). The non-specific adhesion was reduced significantly through treatment with 1% BSA, and the specific adhesion increased steeply with increasing of P-selectin engagement (Fig. 1). A typical real-time dynamic process of calcium signaling of HL-60 at wall shear stresses of 2 dyn/cm^2^, expressing a high shear stress environment in veins vessels [32], was shown in Fig. 2a and b, which indicated that, the cell fluorescence intensity was maintained at lower level for a period of time first, then increased to its peak quickly, and lastly decreased to its initial level gradually. It said that, calcium bursting of a firmly adhered cell occurred after passing through a latent period or delay time (T_D_), which is the time interval between firmly adhering and calcium bursting (Fig. 2b). The peak calcium intensity (I_P_), which was defined as the difference between maximum fluorescence intensity and the averaged fluorescence intensity over delay time, expressed the total release of cytosolic calcium ion (Fig. 2b). The higher the peak calcium intensity, the more the release of cytosolic calcium. This time-course of calcium signaling just with single peak was similar to those in neutrophils [8, 33].

### P-selectin-induced calcium signaling in HL-60 cells was specific and concentration-dependent

**Fig. 3:**
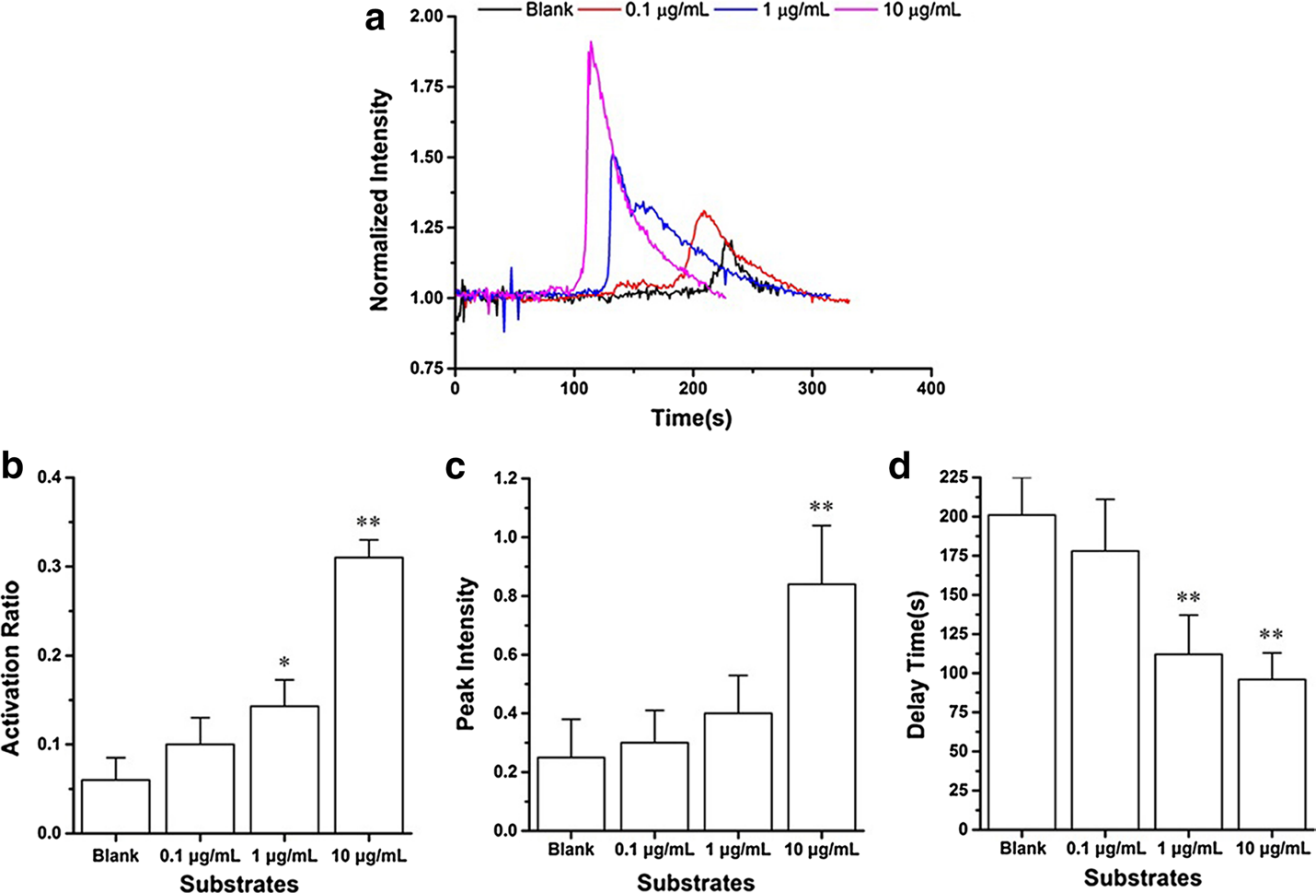
Variation of activation ratio, peak intensity and delay time for calcium signaling of firmly adhered HL-60 under shear stress of 2 dyn/cm^2^ versus immobilized P-selectin concentration. **a** The time-course, **b** the activation ratio, **c** the peak intensity and **d** the delay time of calcium signaling with different of immobilized P-selectin concentrations under wall shear stress of 2 dyn/cm^2^. The data represent the mean plus SEM from at least 20 cells in three experiments. The significant level of difference from blank substrate group was shown by *p* value, * for *p* < 0.05 and ** for *p* < 0.01

We examined the calcium signaling of HL-60 cells on blank (without BSA) and P-selectin (0.1, 1 and 10 μg/mL with BSA)-coated substrates, and measured the cell activation ratio, which was defined as the percentage of calcium signaling cells in all firmly adhered cells under the field of view, to estimate the probability of calcium signaling. The typical time-courses of calcium signaling demonstrated the significant differences between different treatments (Fig. 3a). The activation ratios of HL-60 cells on substrate coated with nothing or BSA just under wall shear stress of 2 dyn/cm^2^ lied in the lowest level in comparison with other three different treated substrates (Fig. 3b), suggesting that the calcium signaling was specifically induced by P-selectin.

The activation ratios of cells on substrates coated with P-selectin of 0.1, 1.0 and 10 μg/mL were equal to 10 ± 3, 14 ± 3 and 31 ± 2%, respectively (Fig. 3b), under wall shear stress of 2 dyn/cm^2^. It said that increasing concentration of P-selectin on substrate enhanced the calcium signaling of HL-60 cells. The peak intensity of calcium signaling increased with the immobilized P-selectin density (Fig. 3c), that is, the more the P-selectin involved in HL-60 adhering, the more the release of cytosolic calcium. It suggested that increasing interaction of P-selectin and PSGL-1 would prompt the cytosolic calcium release of HL-60. The peak intensity of calcium signaling for HL-60 on 10 μg/mL P-selectin were much higher than those for cells on both 0.1 and 1 μg/mL P-selectin, indicating that a sufficient concentration (10 μg/mL) of immobilized P-selectin could induce a significant intracellular calcium flux. And, the delay time of calcium signaling of HL-60 decreased quickly as increasing of the immobilized P-selectin density (Fig. 3d), meaning that increasing interaction of P-selectin and PSGL-1 would quicken the cytosolic calcium release of HL-60. The above data illustrated that the calcium signaling of firmly adhered HL-60 cells was not only specific for P-selectin but also dependent on the P-selectin concentration. A high P-selectin concentration was required for the intensive and quick calcium signaling of HL-60.

### Force triggered and modulated the calcium signaling of HL-60 cells on P-selectin

**Fig. 4:**
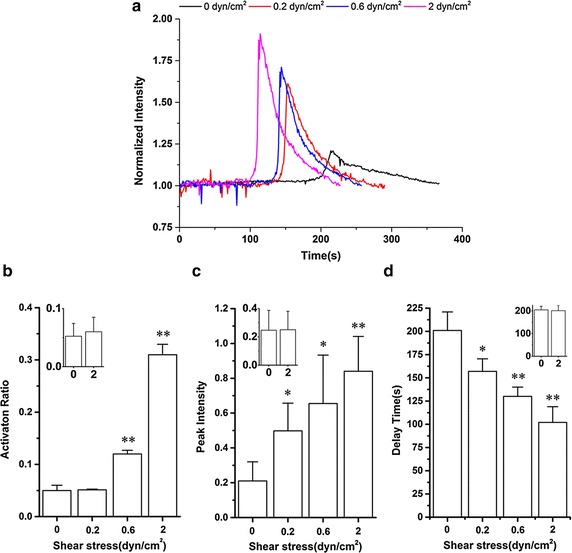
Variation of activation ratio, peak intensity and delay time for calcium signaling of firmly adhered HL-60 against shear stress. The plot illustrates the time-course (**a**), activation ratio (**b**), peak intensity (**c**) and delay time (**d**) for calcium signaling of firmly adhered HL-60 on substrate coated with 10 μg/mL P-selectin under shear stresses of 0.0, 0.2, 0.6 and 2 dyn/cm^2^. The data represent the mean plus SEM from at least 20 cells in three experiments. Each *inset* in **b**, **c** or **d** presents the calcium signaling of HL-60 on blank substrates under shear stresses of 0 and 2 dyn/cm^2^. The significant level of difference from blank substrate group was shown by *p* value, * for *p* < 0.05 and ** for *p* < 0.01

Under various physiological wall shear stresses of 0.0, 0.2, 0.6 and 2.0 dyn/cm^2^, the calcium signaling of HL-60 on substrate coated with 10 μg/mL P-selectin was analyzed to determine the role of mechanical force in this process. The typical time-courses of P-selectin calcium signaling showed a force-regulated calcium bursting in HL-60 (Fig. 4a). It was found that, the calcium bursting was a very weak and rare event under zero shear stress environment, as shown in the very small activation ratio, low calcium peak and long delay time in comparison with those under wall shear stresses ≥0.2 dyn/cm^2^ (Fig. 4b, c, d), meaning that force triggered the P-selectin-induced calcium signaling of HL-60. And, increasing of wall shear stress would upregulate the cell activation ratio or the probability of calcium signaling of HL-60 (Fig. 4b) and rise the peak level of calcium signaling (Fig. 4c) and quicken the cytosolic calcium release of HL-60 (Fig. 4d). These results revealed that mechanical force, as a trigger and regulator for P-selectin-induced calcium signaling of HL-60, was required for significant increment of cytosolic ionized calcium.

In addition, it was shown that the delay time of calcium signaling of HL-60 was 201 ± 20 s at zero force and became 157 ± 13.5 s at 0.2 dyn/cm^2^; as further increasing of the wall shear stress from of 0.2 to 2 dyn/cm^2^, the delay time of calcium signaling was shortened to about 100 s (Fig. 4d). It meant that increasing of wall shear stress would quicken the cytosolic calcium release of HL-60 (Fig. 4d). The insets in Fig. 4b, c and d illustrated that different shear stresses (0 and 2 dyn/cm^2^) had almost same effects on the non-specific calcium signaling of HL-60 (on blank substrate). This phenomenon of force-enhanced calcium signaling may be relevant to the catch bond mechanism of interaction of P-selectin with PSGL-1 [7, 22].

## Discussion

HL-60 cells are principally a type of neutrophilic promyelocyte (precursor) [29]. The regulators of intracellular signaling of HL-60 cells remain unknown. With parallel flow chamber experiment combing with fluorescent detection, we here investigated the mechanical regulation of calcium signaling of HL-60 cells firmly adhered on P-selectin under shear flow, and found that the calcium signaling of HL-60 under flow was induced specifically by immobilized P-selectin and triggered by external force. Mechanical force would cooperate with P-selectin to regulate the calcium signaling of HL-60 cells under flow. Different from the calcium signaling induced by chemokine [17–19], the P-selectin- induced calcium signaling of HL-60 in absence of chemokine required force. It was consistent with the previous report for selectins-induced cytosolic calcium of neutrophils without chemokine under flow [8]. The higher the P-selectin concentration, the more intensive the calcium signaling for HL-60 cells. A possible explanation might be that the more the P-selectin molecules, the more the formed P-selectin/PSGL-1 complex [30, 31], and the more the mechano-chemical message being conveyed into cells.

The P-selectin-induced calcium bursting of HL-60 might be relevant to either release of intracellular calcium stores or influx of extracellular calcium. Perhaps, the P-selecin-induced calcium bursting was referred to a pathway, along which the binding of PSGL-1 to P-selectin not only made cells be capture to substrate [6, 7] but also formed an “Outside-in” channel for mechano-chemical signaling first, and then PSGL-1 activates Src family kinase (SFK) for transmitting signal to downstream signal molecules such as PLC [34]; subsequently, PLC cleaves phosphatidylinositol 4, 5-biphosphate (PIP2) into diglycerides (DAG) and inositol 1,4,5-trisphosphate (IP3) which interacts with IP3 receptor on endoplasmic reticulum (ER) to release the calcium ion from intracellular calcium stores [8, 35–37]. Another signal pathway for P-selectin-induced calcium bursting might be relate to membrane calcium ion channel which was opened in responding the mechanical stretching of P-selectin/PSGL-1 complex like integrins and their ligands [38]. As a result, the extracellular calcium ion would enter into cells, leading to increase of concentration of cytosolic ionized calcium [39].

We found that soluble P-selectin in suspension could not induce calcium signaling of HL-60 (data not shown here) because of that no tensile force acted on P-selectin/PSGL-1 complex, indicating again that force was required for the P-selectin-induced calcium signaling. High wall shear stress (≥0.6 dyn/cm^2^) made P-selectin-induced calcium signaling remarkable (Fig. 4), similar to that E-selectin could increase cytosolic calcium in neutrophils at shear stress of 2 dyn/cm^2^ rather than 0.2 dyn/cm^2^ [8]. Force-enhanced calcium signaling of HL-60 on P-selectin might relevant to tensile force on P-selectin/PSGL-1 complex [40], similar to the calcium signaling mediated by TCR/MHC complex with force-dependent affinity [23]. It suggests that P-selectin/PSGL-1 complex might act as a mechanosensor to activate intracellular downstream signal molecules and further to induce calcium signaling under flow [41]. The present work suggests a novel protocol in research on blood cells. It should be pointed out that our finding was not found in vascular physiological environment because of that selectin mediates rolling rather than firmly adhering of leukocytes, leading underestimate the latency of P-selectin-mediated calcium signaling.

## Conclusion

Here, we demonstrated that the calcium signaling of HL-60 firmly adhered on immobilized P-selectin is P-selectin concentration- and mechanical force-dependent. High immobilized P-selectin concentration and/or external force on cell would make the calcium signaling of cell more intensive. These results might exhibit a novel insight in understanding the mechano-chemical regulation mechanism for intracellular signaling pathways induced by adhesion molecules, and be contributed to some new ideas in risk assessment, clinical diagnosis and the efficacy of inflammation and cancer treatment.
